# Supplementary material for: COVID-19 associated changes in HIV service delivery over time in Central Africa: Results from facility surveys during the first and second waves of the pandemic
Source: PLoS One. 2022 Nov 30;17(11):e0275429. doi: 10.1371/journal.pone.0275429 (PMC9710788; doi:10.1371/journal.pone.0275429)
Supplement: S1 Table — ART: Antiretroviral therapy; IeDEA: International epidemiology Databases to Evaluate AIDS. ** Sites where the service was not available prior to the COVID-19 pandemic excluded from denominator. (DOCX) [file pone.0275429.s001.docx]

**Table S1. Changes in clinic environment or operations at Central Africa IeDEA sites, by country, Round 1 (June - July 2020)**

| **Change in clinic environment or operations** | **Burundi** | **Cameroon** | **Dem. Republic of Congo** | **Republic of Congo** | **Rwanda** | **Total N=21** |
| --- | --- | --- | --- | --- | --- | --- |
| Geographic area surrounding this HIV clinic subject to any form of COVID-19 restrictions on travel, service provision, or business operations | (0%) | (0%) | 1 (100%) | 2 (100%) | 7 (58%) | 10 (48%) |
| Decreases in the number of hours or days of service delivery for HIV patients | (0%) | (0%) | 1 (100%) | 2 (100%) | 5 (42%) | 8 (38%) |
| Reduced availability of HIV care providers | 2 (67%) | 2 (67%) | 1 (100%) | 1 (50%) | 3 (25%) | 9 (43%) |
| Re-assignment of providers to assist with the COVID-19 response | (0%) | 1 (33%) | (0%) | (0%) | 2 (17%) | 3 (14%) |
| COVID-19-related illness, self-isolation, or quarantine | 2 (67%) | 2 (67%) | 1 (100%) | 1 (50%) | 2 (17%) | 8 (38%) |
| Reconfiguration of hospital/clinic space to accommodate COVID-19-related services | 1 (33%) | 2 (67%) | (0%) | 1 (50%) | 4 (33%) | 8 (38%) |
| Increased use of personal protective equipment (masks, gloves, gowns, etc.) by HIV clinic staff | 3 (100%) | 3 (100%) | 1 (100%) | 2 (100%) | 10 (83%) | 19 (91%) |
| Increased use of telemedicine (i.e., consultations by phone/web) in HIV-related care | (0%) | 1 (33%) | 1 (100%) | 1 (50%) | 2 (17%) | 5 (24%) |
| Interruptions or changes in recording of data (either paper or electronic records) related to clinical management of patients | (0%) | (0%) | (0%) | (0%) | 1 (8%) | 1 (5%) |
| Withdrawal/suspension of activities of non-governmental partners that support care provision in the clinic (N=20)** | 0 (0%) | 0 (0%) | 1 (33.3%) | 0 (0%) | 2 (18%) | 3 (15%) |

ART: Antiretroviral therapy; IeDEA: International epidemiology Databases to Evaluate AIDS

** Sites where the service was not available prior to the COVID-19 pandemic excluded from denominator
