# Supplementary material for: COVID-19 associated changes in HIV service delivery over time in Central Africa: Results from facility surveys during the first and second waves of the pandemic
Source: PLoS One. 2022 Nov 30;17(11):e0275429. doi: 10.1371/journal.pone.0275429 (PMC9710788; doi:10.1371/journal.pone.0275429)
Supplement: S2 Table — ART: Antiretroviral therapy; IeDEA: International epidemiology Databases to Evaluate AIDS; PrEP: Pre-exposure prophylaxis. ** Sites where the service was not available prior to the COVID-19 pandemic excluded from denominator. (DOCX) [file pone.0275429.s002.docx]

**Table S2. Changes in clinic environment or operations at Central Africa IeDEA sites, by country, Round 2 (October 2020 - February 2021)**

| **Change in clinic environment or operations** | **Burundi** | **Cameroon** | **Dem. Republic of Congo** | **Republic of Congo** | **Rwanda** | **Total N=21** |
| --- | --- | --- | --- | --- | --- | --- |
| Geographic area surrounding this HIV clinic subject to any form of COVID-19 restrictions on travel, service provision, or business operations | 1 (33%) | 1 (33%) | 1 (100%) | 2 (100%) | 10 (83%) | 15 (71.4%) |
| Duration of lockdowns/restrictions (as of 2nd survey) |  |  |  |  |  |  |
| ≤1 month | (0%) | (0%) | (0%) | (0%) | 5 (42%) | 5 (24%) |
| 2-3 months | (0%) | 1 (33%) | (0%) | 2 (100%) | 3 (25%) | 6 (29%) |
| 5+ months or ongoing | (0%) | (0%) | 1 (100%) | (0%) | 1 (8%) | 2 (10%) |
| Do not know/recall | 1 (33%) | (0%) | (0%) | (0%) | 1 (8%) | 2 (10%) |
| Not applicable | 2 (67%) | 2 (67%) | (0%) | (0%) | 2 (17%) | 6 (29%) |
| Decreases in the number of hours or days of service delivery for HIV patients | (0%) | (0%) | (0%) | (0%) | 1 (8%) | 1 (5%) |
| Reduced availability of HIV care providers | (0%) | 1 (33%) | (0%) | (0%) | 4 (33%) | 5 (24%) |
| Re-assignment of providers to assist with the COVID-19 response | (0%) | 1 (33%) | (0%) | (0%) | 2 (17%) | 3 (14%) |
| COVID-19-related illness, self-isolation, or quarantine | (0%) | (0%) | (0%) | (0%) | 3 (25%) | 3 (14%) |
| Reconfiguration of hospital/clinic space to accommodate COVID-19-related services | 1 (33%) | 2 (67%) | (0%) | (0%) | 4 (33%) | 7 (33%) |
| Increased use of personal protective equipment (masks, gloves, gowns, etc.) by HIV clinic staff | 2 (67%) | 3 (100%) | 1 (100%) | 1 (50%) | 9 (75%) | 16 (76%) |
| Increased use of telemedicine (i.e., consultations by phone/web) in HIV-related care | (0%) | 2 (67%) | (0%) | (0%) | 2 (17%) | 4 (19%) |
| Interruptions or changes in recording of data (either paper or electronic records) related to clinical management of patients | (0%) | 1 (33%) | (0%) | (0%) | 1 (8%) | 2 (10%) |
| Withdrawal/suspension of activities of non-governmental partners that support care provision in the clinic (N=20)** | (0%) | (0%) | (0%) | (0%) | (0%) | (0%) |

ART: Antiretroviral therapy; IeDEA: International epidemiology Databases to Evaluate AIDS; PrEP: Pre-exposure prophylaxis

** Sites where the service was not available prior to the COVID-19 pandemic excluded from denominator
