# Supplementary material for: COVID-19 associated changes in HIV service delivery over time in Central Africa: Results from facility surveys during the first and second waves of the pandemic
Source: PLoS One. 2022 Nov 30;17(11):e0275429. doi: 10.1371/journal.pone.0275429 (PMC9710788; doi:10.1371/journal.pone.0275429)
Supplement: S4 Table — ART: Antiretroviral therapy; IeDEA: International epidemiology Databases to Evaluate AIDS; PrEP: Pre-exposure prophylaxis. ** Sites where the service was not available prior to the COVID-19 pandemic excluded from denominator. (DOCX) [file pone.0275429.s004.docx]

**Table S4. Effects of COVID-19 on HIV-related services and capacity at Central Africa IeDEA sites, by country, Round 2 (Oct 2020 - Feb 2021)**

| **Effects of COVID-19 on HIV-related services and programs** | **Burundi** | **Cameroon** | **D. R. of Congo** | **Republic of Congo** | **Rwanda** | **Total N=21** |
| --- | --- | --- | --- | --- | --- | --- |
| **HIV testing and care enrollment** |  |  |  |  |  |  |
| Suspension or decreases in HIV testing/diagnostic services | (0%) | (0%) | (0%) | (0%) | (0%) | (0%) |
| Suspension or postponement of the enrollment of new patients in HIV care | (0%) | (0%) | (0%) | (0%) | (0%) | (0%) |
| Suspension or postponement of non-urgent appointments for HIV patients | (0%) | (0%) | (0%) | (0%) | (0%) | (0%) |
| **ART services** |  |  |  |  |  |  |
| ART clinics have been suspended or shut down | (0%) | (0%) | (0%) | (0%) | (0%) | (0%) |
| ART initiation services have been suspended | (0%) | (0%) | (0%) | (0%) | (0%) | (0%) |
| Patients given extra supplies/refills of ART to reduce the frequency of refills | 3 (100%) | 2 (67%) | (0%) | 2 (100%) | 8 (67%) | 15 (71%) |
| ART pick-up points have been designated in the community | (0%) | (0%) | (0%) | (0%) | (0%) | (0%) |
| Expansion of same-day/rapid ART initiation | 1 (33%) | 2 (67%) | (0%) | (0%) | 2 (17%) | 5 (24%) |
| Adherence counseling streamlined | (0%) | (0%) | (0%) | (0%) | 2 (17%) | 2 (10%) |
| **Viral load testing services** |  |  |  |  |  |  |
| Sample collection suspended | (0%) | (0%) | (0%) | (0%) | 1 (8%) | 1 (5%) |
| VL samples no longer accepted | (0%) | (0%) | (0%) | (0%) | 1 (8%) | 1 (5%) |
| Longer turn-around time | 1 (33%) | (0%) | (0%) | (0%) | 4 (33%) | 5 (24%) |
| Other (staffing shortages, lack of transport for samples) | (0%) | (0%) | (0%) | (0%) | (0%) | (0%) |
| **Community-based services** |  |  |  |  |  |  |
| Suspension of activities of NGO partners that support community-based programs for patients enrolled in HIV care at the clinic | 0 (0%) | 0 (0%) | 0 (0%) | 0 (0%) | 0 (0%) | 0 (0%) |
| Community-based HIV testing suspended (N=17)** | (0%) | (0%) | (0%) | - | 3 (30%) | 3 (18%) |
| Community-based ART refills suspended (N=18)** | (0%) | (0%) | (0%) | (0%) | 1 (10%) | 1 (6%) |
| Community-based support group meetings/activities suspended (N=20)** | (0%) | (0%) | (0%) | (0%) | 4 (36%) | 4 (20%) |
| Community-based tracing of patients who are lost to follow-up (LTFU) suspended | (0%) | (0%) | (0%) | (0%) | 1 (8%) | 1 (5%) |
| **Stockouts** |  |  |  |  |  |  |
| PrEP | (0%) | (0%) | (0%) | (0%) | 1 (8%) | 1 (5%) |
| HIV test kits | 1 (33%) | (0%) | (0%) | 1 (50%) | (0%) | 2 (10%) |
| First-line ART | (0%) | (0%) | (0%) | (0%) | (0%) | (0%) |
| Second-line ART | (0%) | 1 (33%) | (0%) | (0%) | (0%) | 1 (5%) |
| Third-line ART (N=19)** | 1 (33%) | (0%) | - | 1 (50%) | 1 (9%) | 3 (16%) |
| Supplies for viral load testing | (0%) | (0%) | (0%) | (0%) | 5 (42%) | 5 (24%) |

ART: Antiretroviral therapy; IeDEA: International epidemiology Databases to Evaluate AIDS; PrEP: Pre-exposure prophylaxis

** Sites where the service was not available prior to the COVID-19 pandemic excluded from denominator
