# Supplementary material for: COVID-19 associated changes in HIV service delivery over time in Central Africa: Results from facility surveys during the first and second waves of the pandemic
Source: PLoS One. 2022 Nov 30;17(11):e0275429. doi: 10.1371/journal.pone.0275429 (PMC9710788; doi:10.1371/journal.pone.0275429)
Supplement: S1 File — (DOCX) [file pone.0275429.s005.docx]

**Supporting Information: Acknowledgements**

The authors would like to thank the following site investigators, clinicians and data managers who distributed and completed the 2020 IeDEA Site Assessment, along with members of the IeDEA Site Assessment Working Group.

**CA-IeDEA Cohorts and Site investigators:**

Nimbona Pélagie, Association Nationale de Soutien aux Séropositifs et Malade du Sida (ANSS), Burundi; Patrick Gateretse, Jeanine Munezero, Valentin Nitereka, Théodore Niyongabo, Christelle Twizere, Centre National de Référence en Matière de VIH/SIDA, Burundi; Hélène Bukuru, Thierry Nahimana, Centre de Prise en Charge Ambulatoire et Multidisciplinaire des PVVIH/SIDA du Centre Hospitalo-Universitaire de Kamenge (CPAMP-CHUK), Burundi; Elysée Baransaka, Patrice Barasukana, Eugene Kabanda, Martin Manirakiza, François Ndikumwenayo, CHUK/Burundi National University, Burundi; Jérémie Biziragusenyuka, Ange Marie Michelline Munezero, Centre de Prise en Charge Ambulatoire et Multidisciplinaire des PVVIH/SIDA de l’Hôpital Prince Régent Charles (CPAMP-HPRC), Burundi; Denis Nsame Nforniwe, Bamenda Hospital, Cameroon; Rogers Ajeh, Marc Lionel Ngamani, Clinical Research Education and Consultancy (CRENC), Cameroon; Anastase Dzudie, CRENC and Douala General Hospital, Cameroon; Akindeh Mbuh, CRENC and University of Yaoundé, Cameroon; Djenabou Amadou, Eric Walter Pefura Yone, Jamot Hospital, Cameroon; Ernestine Kendowo, Limbe Regional Hospital, Cameroon; Catherine Akele, Akili Clever, Faustin Kitetele, Patricia Lelo, Martine Tabala, Kalembelembe Pediatric Hospital, Democratic Republic of Congo; Cherubin Ekembe, Didine Kaba, Kinshasa School of Public Health, Democratic Republic of Congo; Merlin Diafouka, Martin Herbas Ekat, Dominique Mahambou Nsonde, CTA Brazzaville, Republic of Congo; Adolphe Mafoua, Massamba Ndala Christ, CTA Pointe-Noire, Republic of Congo; Jules Igirimbabazi, Nicole Ayinkamiye, Bethsaida Health Center, Rwanda; Providance Uwineza, Emmanuel Ndamijimana, Busanza Health Center, Rwanda; Emmanuel Habarurema, Marie Luise Nyiraneza, Gahanga Health Center, Rwanda; Marie Louise Nyiransabimana, Liliane Tuyisenge, Gikondo Health Center, Rwanda; Christian Shyaka, Catherine Kankindi, Kabuga Health Center, Rwanda; Bonheur Uwakijijwe, Marie Grace Ingabire, Kicukiro Health Center, Rwanda; Jules Ndumuhire, Marie Goretti Nyirabahutu, Masaka Health Center, Rwanda; Fred

Muyango, Jean Christophe Bihibindi, Nyagasambu Health Center, Rwanda; Oliver Uwamahoro, Yvette Ndoli, Nyarugunga Health Center, Rwanda; Sabin Nsanzimana, Placidie Mugwaneza, Eric Remera, Esperance Umumararungu, Gallican Nshogoza Rwibasira, Dominique Savio Habimana, Rwanda Biomedical Center, Rwanda; Josephine Gasana, Faustin Kanyabwisha, Gallican Kubwimana, Benjamin Muhoza, Athanase Munyaneza, Gad Murenzi, Francoise Musabyimana, Francine Umwiza, Charles Ingabire, Patrick Tuyisenge, Alex M. Butera, Jules Kabahizi, Ephrem Rurangwa, Rwanda Military Hospital, Rwanda; Rosine Feza, Eugenie Mukashyaka, Shyorongi Health Center, Rwanda; Chantal Benekigeri, Jacqueline Musaninyange, WE-ACTx Health Center, Rwanda.

**Coordinating and Data Centers:**

Adebola Adedimeji, Kathryn Anastos, Madeline Dilorenzo, Lynn Murchison, Jonathan Ross, Marcel Yotebieng, Albert Einstein College of Medicine, USA; Diane Addison, Ellen Brazier, Heidi Jones, Elizabeth Kelvin, Sarah Kulkarni, Denis Nash, Matthew Romo, Olga Tymejczyk, Institute for Implementation Science in Population Health, Graduate School of Public Health and Health Policy, City University of New York (CUNY), USA; Batya Elul, Columbia University, USA; Xiatao Cai, Allan Dong, Don Hoover, Hae-Young Kim, Chunshan Li, Qiuhu Shi, Data Solutions, USA; Kathryn Lancaster, The Ohio State University, USA; Mark Kuniholm, University at Albany, State University of New York, USA; Andrew Edmonds, Angela Parcesepe, Jess Edwards, University of North Carolina at Chapel Hill, USA; Olivia Keiser, University of Geneva; Stephany Duda; Vanderbilt University School of Medicine, USA; April Kimmel, Virginia Commonwealth University School of Medicine, USA.

**Funding information**

Research reported in this publication was supported by the National Institutes of Health’s National Institute of Allergy and Infectious Diseases (NIAID), the Eunice Kennedy Shriver National Institute of Child Health & Human Development (NICHD), the National Cancer Institute (NCI), the National Institute on Drug Abuse (NIDA), the National Heart, Lung, and Blood Institute (NHLBI), the National Institute on Alcohol Abuse and Alcoholism (NIAAA), the National Institute of Diabetes and Digestive and Kidney Diseases (NIDDK), the Fogarty International Center (FIC), the National Library of Medicine (NLM), and the Office of the Director (OD) under Award Number U01AI096299 (Central Africa-IeDEA). The content is solely the responsibility of the authors and does not necessarily represent the official views of the National Institutes of Health. The funders, however, had no role in study design, data collection and analysis, decision to publish, or preparation of the manuscript.
