# Supplementary material for: COVID-19 associated changes in HIV service delivery over time in Central Africa: Results from facility surveys during the first and second waves of the pandemic
Source: PLoS One. 2022 Nov 30;17(11):e0275429. doi: 10.1371/journal.pone.0275429 (PMC9710788; doi:10.1371/journal.pone.0275429)
Supplement: Attachment — Submitted filename: Response to reviewers_06292022 (1).docx [file pone.0275429.s006.docx]

Journal Requirements:

When submitting your revision, we need you to address these additional requirements.

**1. Please ensure that your manuscript meets PLOS ONE's style requirements, including those for file naming. The PLOS ONE style templates can be found at**

[https://journals.plos.org/plosone/s/file?id=wjVg/PLOSOne_formatting_sample_main_body.pdf](https://urldefense.proofpoint.com/v2/url?u=https-3A__journals.plos.org_plosone_s_file-3Fid-3DwjVg_PLOSOne-5Fformatting-5Fsample-5Fmain-5Fbody.pdf&d=DwMFaQ&c=mRWFL96tuqj9V0Jjj4h40ddo0XsmttALwKjAEOCyUjY&r=zhp8Wa8jSo-Azo9A9FPm7ePTAz2zucw4miM28EKxb38&m=J1Aw-3L5Ys1AQO_asbv-LClQTQ37AA5ybXp05EiuhLw&s=oFh5S5qARc8FfF6ERZRu4GePhcTmmR-TgWeRAyhRURM&e=)  and

[https://journals.plos.org/plosone/s/file?id=ba62/PLOSOne_formatting_sample_title_authors_affiliations.pdf](https://urldefense.proofpoint.com/v2/url?u=https-3A__journals.plos.org_plosone_s_file-3Fid-3Dba62_PLOSOne-5Fformatting-5Fsample-5Ftitle-5Fauthors-5Faffiliations.pdf&d=DwMFaQ&c=mRWFL96tuqj9V0Jjj4h40ddo0XsmttALwKjAEOCyUjY&r=zhp8Wa8jSo-Azo9A9FPm7ePTAz2zucw4miM28EKxb38&m=J1Aw-3L5Ys1AQO_asbv-LClQTQ37AA5ybXp05EiuhLw&s=m5rvOwLSunKIRYX_-SgiDnG4siw8CAO6P0RkND6i6tI&e=)

We thank the Editor for considering our manuscript. In our revised manuscript, we have aligned the formatting of the manuscript and file names with PLOS ONE’s style templates.

**2. Please provide additional details regarding participant consent. In the ethics statement in the Methods and online submission information, please ensure that you have specified (1) whether consent was informed and (2) what type you obtained (for instance, written or verbal, and if verbal, how it was documented and witnessed). If your study included minors, state whether you obtained consent from parents or guardians. If the need for consent was waived by the ethics committee, please include this information.**

**If you are reporting a retrospective study of medical records or archived samples, please ensure that you have discussed whether all data were fully anonymized before you accessed them and/or whether the IRB or ethics committee waived the requirement for informed consent. If patients provided informed written consent to have data from their medical records used in research, please include this information.**

We appreciate the Editor’s feedback and advice. We have clarified that the study was determined to be non-human subjects research for which informed consent was not required (lines 165-172).

**3. Thank you for stating the following in the Acknowledgments Section of your manuscript:**

**“Research reported in this publication was supported by the National Institutes of Health’s National Institute of Allergy and Infectious Diseases (NIAID), the Eunice Kennedy Shriver National Institute of Child Health & Human Development (NICHD), the National Cancer Institute (NCI), the National Institute on Drug Abuse (NIDA), the National Heart, Lung, and Blood Institute (NHLBI), the National Institute on Alcohol Abuse and Alcoholism (NIAAA), the National Institute of Diabetes and Digestive and Kidney Diseases (NIDDK), the Fogarty International Center (FIC), the National Library of Medicine (NLM), and the Office of the Director (OD) under Award Number U01AI096299 (Central Africa-IeDEA). The content is solely the responsibility of the authors and does not necessarily represent the official views of the National Institutes of Health.”**

**Please note that funding information should not appear in the Acknowledgments section or other areas of your manuscript. We will only publish funding information present in the Funding Statement section of the online submission form**.

Thank you for clarifying these points. We have removed the funding information from the Acknowledgements section. For readability we have also shortened the Acknowledgements and presented the full list of sites and individuals involved as Supplementary Information.

**Please remove any funding-related text from the manuscript and let us know how you would like to update your Funding Statement. Currently, your Funding Statement reads as follows:**

**“Research reported in this publication was supported by the National Institutes of Health’s National Institute of Allergy and Infectious Diseases (NIAID), the Eunice Kennedy Shriver National Institute of Child Health & Human Development (NICHD), the National Cancer Institute (NCI), the National Institute on Drug Abuse (NIDA), the National Heart, Lung, and Blood Institute (NHLBI), the National Institute on Alcohol Abuse and Alcoholism (NIAAA), the National Institute of Diabetes and Digestive and Kidney Diseases (NIDDK), the Fogarty International Center (FIC), the National Library of Medicine (NLM), and the Office of the Director (OD) under Award Number U01AI096299 (Central Africa-IeDEA). The content is solely the responsibility of the authors and does not necessarily represent the official views of the National Institutes of Health”**

**Please include your amended statements within your cover letter; we will change the online submission form on your behalf.**

We have removed all funding-related text from the manuscript and confirm that no changes are needed to the funding statement.

**4. Please include your full ethics statement in the ‘Methods’ section of your manuscript file. In your statement, please include the full name of the IRB or ethics committee who approved or waived your study, as well as whether or not you obtained informed written or verbal consent. If consent was waived for your study, please include this information in your statement as well.**

As noted above, we have added the full name of the IRB which determined that the study was non-human subjects research for which informed consent was not required (lines 165-172).

**Please review your reference list to ensure that it is complete and correct. If you have cited papers that have been retracted, please include the rationale for doing so in the manuscript text, or remove these references and replace them with relevant current references. Any changes to the reference list should be mentioned in the rebuttal letter that accompanies your revised manuscript. If you need to cite a retracted article, indicate the article’s retracted status in the References list and also include a citation and full reference for the retraction notice.

Additional Editor Comments (if provided):

Your paper was of interest to the reviewers. As you can see below, the reviewers identified both strengths and weaknesses in your paper. The reviewers noted important areas of your paper that require careful attention. From my own reading of your paper, I am in agreement with the reviewers that your paper will make an important contribution and that your paper will benefit from a thoughtful revision. Please revise your paper in accordance with the reviews. In particular please review for grammar and punctuation errors and include lessons learned from intra and inter country variations as also mentioned by reviewer 1.

Please be sure to include a cover letter with your revision that addresses all of the reviews and comments as well as my comments, point-by-point. Your revision should also carefully follow the journal style as presented in the Instructions for Authors available at the journal website.**

Dear Editor,

Thank you for your consideration and feedback on our paper. We have worked to address each of the reviewers’ suggestions in our revision, and have addressed the reviewers’ feedback point by point, below. All line numbers refer to the clean version of the manuscript. We have also made additional edits, in response to your suggestions to align the manuscript with the journal requirements, while also editing the manuscript to improve the readability of the manuscript and address grammar and punctuation errors.

Sincerely,

Rogers Ajeh

**Responses to reviewers’ comments**

**Reviewer 1**

1. **Key results in the abstract and the text allude to negative outcomes related to HIV service delivery in light of the pandemic, such as stockouts of HIV testing commodities and antiretroviral treatments (ART), in particular during the second, follow up survey. However much less is discussed in terms of strategies adopted for mitigation of these negative outcomes, if any. It ably dwells on consequences, and less on changes effected, or adaptation outcomes. This is important for establishing what worked and drawing lessons for the future in these and/ or in other countries similarly affected.**

Thank you very much for this comment. We had noted in our abstract that more sites reported expanding rapid ART initiation services and providing extra supplies of ART medications, however, we had not characterized these changes as mitigation strategies. In our revision, we have explicitly described these practices as mitigation strategies (lines 33-35). We have also added a statement about the proportion of sites reporting increased use of telemedicine during the first survey (line 32).

2. **While disruptions due to the pandemic, as noted, are important, more attempt should be made to not only document, but also explain any differences observed, between and within the countries studied. Lessons can also be drawn from any inter and intra country variations in responses adopted to mitigate consequences of the COVID-19 pandemic. This thus takes full advantage of the multi-country approach.**

Thank you for this comment. As noted among our study limitations, the clinics participating in IeDEA may not be representative of HIV clinics in each country, and findings may reflect recall errors, particularly as there were numerous and frequent changes in national policy responses during the first year of the COVID-19 pandemic.^1^ Because of our small sample size and these limitations, we are hesitant to over-interpret some of the observed variations within and between countries reflected in our study. That said, we have given more attention to some of the differences observed between countries in presenting the results, and we have highlighted them further in the discussion—noting in particular that some changes may reflect the easing of government restrictions in settings, such as Rwanda, ROC, and DRC, which had more stringent containment measures early in the pandemic than Burundi and Cameroon (lines 269-272).

**Reviewer 2**

1. **Did all of the site characteristics remain the same pre and during the pandemic?**

We appreciate the reviewer’s consideration of our manuscript and feedback. While we sought to assess pandemic-related impacts on HIV service delivery, we did not attempt to document changes in site characteristics, such as health facility level, urban vs. rural location, population served, and other characteristics reported in Table 1. As we note in our Methods section (lines 107-110), survey data were linked with existing descriptive information about participating sites—data that are stored in a separate database. Although IeDEA does update this database on participating sites every two to three years, site-level characteristics, such as facility designation, urbanicity, or patient population rarely change in these settings, and the interval of time between the two study surveys was very short. For characteristics related to ART initiation and viral load testing practices, we explored practices in 2019, prior to the start of the COVID-19 pandemic, as a reference point for understanding how practices changed during the first year of the pandemic.

2. **You mentioned that the survey captured, "...issuance and timing of any government restrictions and public health measures to limit the spread of COVID-19 in the locality of each clinic". Can you add any more of this information to the manuscript?**

Thank you for raising this point. As described in Table 2 and lines 147-148, at each survey timepoint, we explored whether sites’ locality or area of coverage had been subject to restrictions on travel, service provision or business operations because of the pandemic, and we sought to estimate the duration of these restrictions. We did not attempt to document different types of restrictions or public health measures because we were concerned about recall errors during a period of frequent changes in public health policy and pandemic mitigation measures. To avoid the perception that we aimed to document specific types of government restrictions or public health measures, we have modified the wording in our Methods section (lines 100-101), explaining that the survey gathered information on whether sites’ localities “had been subject to any government restrictions or public health measures to limit the spread of COVID-19.”

3. **In Table 3, I wouldn't label the title "Effects" given you aren't actually showing a causal relationship here in your table. Please identify other words that don't have the same connotation.**

Thank you for this suggestion. We have modified the table title, changing it to: Changes in HIV-related services and capacity at Central Africa IeDEA sites during the first- and second round surveys, 2020-2021.

4. **In your tables, try and provide more space in your rows so the data can more easily be read; perhaps shrink the font.**

Thank you for this suggestion. We have reduced the font size for each table and increased the spacing. Additionally, to improve readability and to be consistent with similar site survey research^2^ published in PLoS One, we have removed the decimal points in reporting percentages throughout both the manuscript and all tables.

References

1.	Coronavirus Pandemic (COVID-19). OurWorldInData.org; 2020. <https://ourworldindata.org/coronavirus>. Accessed June 27, 2022.

2.	Parcesepe AM, Lancaster K, Edelman EJ, et al. Substance use service availability in HIV treatment programs: Data from the global IeDEA consortium, 2014-2015 and 2017. *PLoS ONE.* 2020;15(8):e0237772.
